# Mining key circRNA-associated-ceRNA networks for milk fat metabolism in cows with varying milk fat percentages

**DOI:** 10.1186/s12864-024-10252-y

**Published:** 2024-04-01

**Authors:** Xiaofang Feng, Lijia Tong, Lina Ma, Tong Mu, Baojun Yu, Ruoshuang Ma, Jiwei Li, Chuanchuan Wang, Juan Zhang, Yaling Gu

**Affiliations:** 1 Key Laboratory of Ruminant Molecular and Cellular Breeding, School of Agriculture, Ningxia University, 750021 Yinchuan, China; 2 NingXia Academy of Agriculture and Forestry Sciences, 750002 Yinchuan, China; 3 School of Life Science, Yan’an University, 716000 Yanan, China

**Keywords:** Dairy cows, circRNAs, Milk fat percentage, WGCNA, ceRNAs

## Abstract

**Background:**

Cow milk fat is an essential indicator for evaluating and measuring milk quality and cow performance. Growing research has identified the molecular functions of circular RNAs (circRNAs) necessary for mammary gland development and lactation in mammals.

**Method:**

The present study analyzed circRNA expression profiling data in mammary epithelial cells (MECs) from cows with highly variable milk fat percentage (MFP) using differential expression analysis and weighted gene co-expression network analysis (WGCNA).

**Results:**

A total of 309 differentially expressed circRNAs (DE-circRNAs) were identified in the high and low MFP groups. WGCNA analysis revealed that the pink module was significantly associated with MFP (*r* = − 0.85, *P* = 0.007). Parental genes of circRNAs in this module were enriched mainly in lipid metabolism-related signaling pathways, such as focal adhesion, ECM-receptor interaction, adherens junction and AMPK. Finally, six DE-circRNAs were screened from the pink module: circ_0010571, circ_0007797, circ_0002746, circ_0003052, circ_0004319, and circ_0012840. Among them, circ_0002746, circ_0003052, circ_0004319, and circ_0012840 had circular structures and were highly expressed in mammary tissues. Subcellular localization revealed that these four DE-circRNAs may play a regulatory role in the mammary glands of dairy cows, mainly as competitive endogenous RNAs (ceRNAs). Seven hub target genes (*GNB1, GNG2, PLCB1, PLCG1, ATP6V0C, NDUFS4*, and *PIGH*) were obtained by constructing the regulatory network of their ceRNAs and then analyzed by CytoHubba and MCODE plugins in Cytoscape. Functional enrichment analysis revealed that these genes are crucial and most probable ceRNA regulators in milk fat metabolism.

**Conclusions:**

Our study identified several vital circRNAs and ceRNAs affecting milk fat synthesis, providing new research ideas and a theoretical basis for cow lactation, milk quality, and breed improvement.

**Supplementary Information:**

The online version contains supplementary material available at 10.1186/s12864-024-10252-y.

## Introduction

Milk fat an important nutrient and key evaluation indicator for milk. Conjugated linoleic acid—which is abundant in milk fat—is essential for cholesterol downregulation [1] and low-density lipoprotein levels [2] in humans and for the defense against atherosclerosis [3]. Milk fat contains essential minerals and fat-soluble vitamins for humans [4–6]. Cheese—a further processed milk product—holds a significant position in the global premium market for dairy products. However, milk fat has an important influence on cheese flavor, not only because the fatty acids used to synthesize cheese are flavor substances in their own right [7], but also as precursors to flavor substances, such as methyl ketones, secondary alcohols, lactones, and esters, in cheese [8]. Therefore, the safe and effective increase of milk fat content in milk is one of the necessary tools to strengthen the dairy industry’s core competitiveness in the global market.

The advancement of sequencing technology and bioinformatics algorithms has enabled the identification of a class of potentially functional circular RNAs (circRNAs) [9]. These circRNAs are linked together through the 5’ and 3’ ends of the parent gene to form a circular structure [10]. Studies have demonstrated that circRNAs have important molecular functions for mammary gland development and lactation in mammals. circRNA-006258 is closely related to mammary epithelial cell (MEC) growth and milk synthesis in goats [11]. circ_015343 reduces milk production and milk fat synthesis and inhibits MEC growth in sheep [12]. circ01592 and circ09863 increase the levels of triglycerides, cholesterol and unsaturated fatty acids content in MECs of dairy cows [13–14]. circRNA8220 promotes the proliferation and synthesis of β-casein and triglycerides in the MECs of goat [15]. Considering the importance of circRNAs to MECs, we discovered more circRNAs affecting MEC growth and lactation using differential expression analysis and weighted gene co-expression network analysis (WGCNA). WGCNA is an effective systems biology method for analyzing RNA-seq data, including mRNA [16–17], miRNA [18–19], lncRNA [20–21], and circRNA [22–23]. WGCNA canlocate core genes faster using gene connectivity information. Weak effector genes can also be mined, elucidating the biological mechanisms underlying traits. The differential expression analysis and WGCNA methods complement each other and help in the rapid and comprehensive identification of the DE-circRNAs that regulate MFP.

Given the potential of circRNAs to indirectly regulate gene expression in MECs, it is necessary to identify and characterize circRNAs in MECs of cows with different milk fat percentage (MFP), since this circRNAs may be involved in epigenetic and genetic regulation of mammary function. In this study, RNA-seq was used to obtain the expression levels of circRNA in MECs of lactating cows with significantly differential MFP. DE-circRNAs mediating milk lipid metabolism were mined by using differential expression analysis and WGCNA, and we explore the effects of circRNA-mediated regulatory networks on mammary gland development and lactation in dairy cows. This study provides a new research idea and a theoretical basis for future studies on the mechanism of circRNA-regulated milk fat metabolism in dairy cows.

## Results

### Combined differential expression analysis and WGCNA screening for candidate DE-circRNAs

**Fig. 1:**
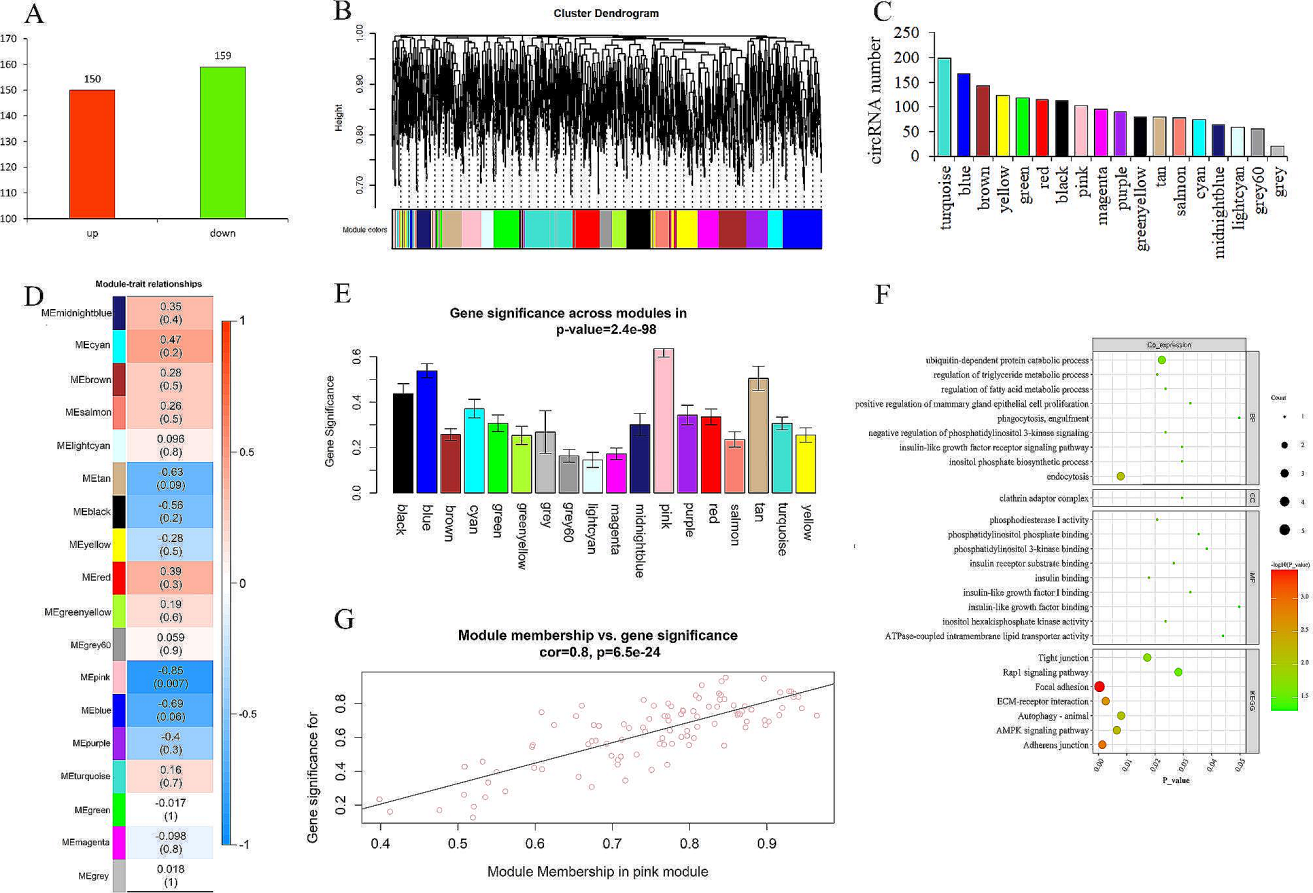
Differential expression analysis and WGCNA of circRNAs in MECs of cows with different MFP. (**A**) DE-circRNAs in the HMF and LMF groups. (**B**) Clustered tree diagram of circRNAs in MECs of dairy cows, each color represents a module. (**C**) The number of circRNAs clustered in different modules. (**D**) Heatmap of correlation between modules and MFP (each module contains correlation coefficient and corresponding *P*-value). (**E**) Module significance values ​​for co-expression modules associated with MFP (module significance values ​​represent a summary of circRNA significance for all circRNAs in each module, with different column colors representing different modules). (**F**) Functional annotation of circRNAs in the pink module. (**G**) A scatterplot of GS for MFP vs. MM in the pink module respectively (a dot represents a circRNA in the pink module)

We constructed a circRNA library of MECs from the high- and low-MFP groups, then sequenced and identified circRNAs. A total of 309 DE-circRNAs were found at a significantly higher level in the high-MFP group than in the low-MFP group (Fig. 1A; Table S1). Subsequently, WGCNA was constructed using transcriptome sequencing data. A total of 18 co-expression modules were obtained after merging modules with a similarity more significant than 75% (Fig. 1B). The number of circRNAs contained in each module ranged from 20 (grey) to 198 (turquoise), among which there were eight modules with over 100 circRNAs, including the turquoise, blue, brown, and yellow modules (Fig. 1C). Module-trait correlation analysis demonstrated that multiple modules were associated with MFP (Fig. 1D), among which the pink module was significantly negatively correlated with MFP (*r* = − 0.85, *P* = 0.007) (Fig. 1D-E). The pink module contained 101 circRNAs (Table S2). Functional annotation of 101 circRNAs revealed that the significantly enriched GO terms included regulation of the triglyceride metabolic process, regulation of the fatty acid metabolic process, positive regulation of MEC proliferation, and the relation of insulin and phosphatidylinositol. The significantly enriched Kyoto Encyclopedia of Genes and Genomes (KEGG) pathways included focal adhesion, ECM-receptor interaction, and adherens junction. These findings suggests that the pink module might contain critical circRNAs that regulate lipid metabolism (Fig. 1F). Subsequently, the gene significance (GS) and module membership (MM) values were calculated to screen the key circRNAs (Fig. 1G), resulting in 11 key circRNAs (|GS| ≥ 0.90 and|MM| ≥ 0.60). Among them, circ_0010571, circ_0007797, circ_0002746, circ_0003052, circ_0004319, and circ_ 0012840 belonged to DE-circRNAs (Table S3); therefore, these circRNAs were further validated as candidates.

### Validation of the circular structure of candidate circRNAs

**Fig. 2:**
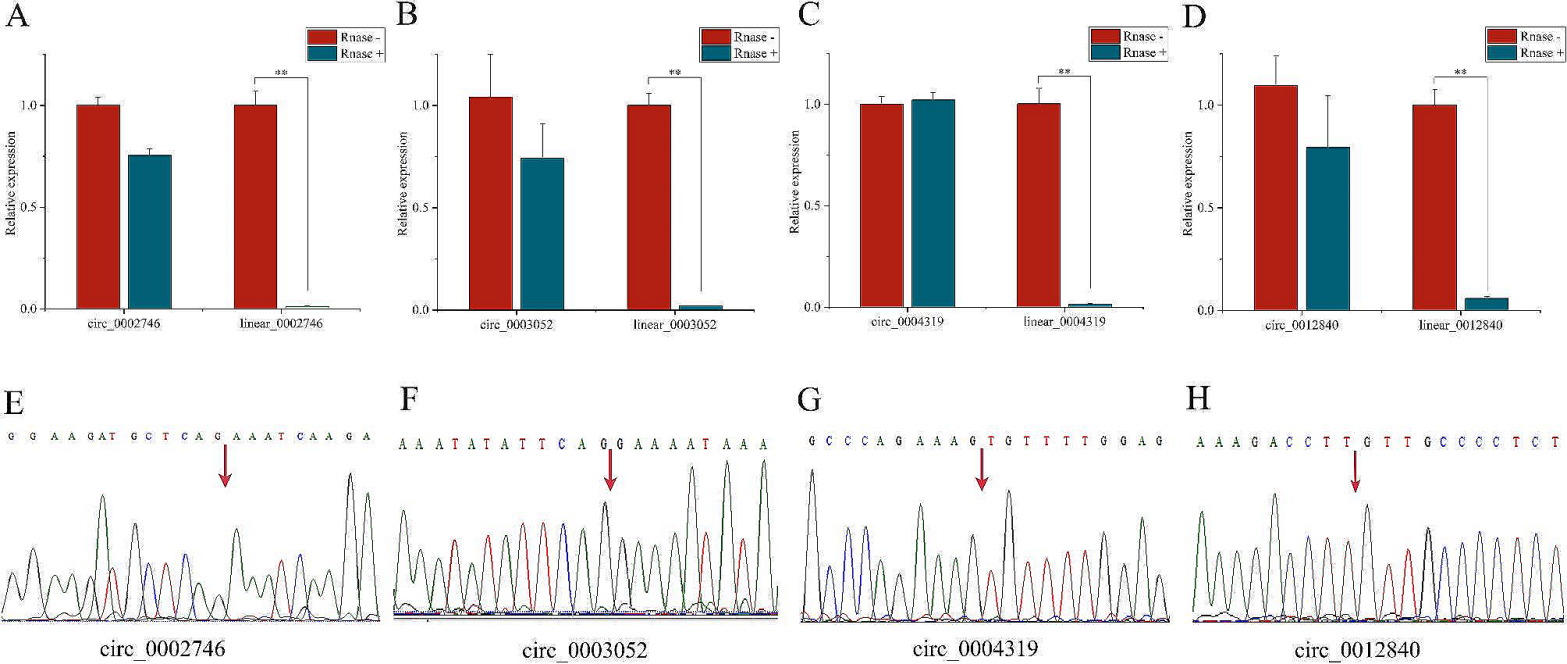
Circular structure identification of circRNAs. (**A**-**D**) We identified by RT-qPCR using divergent and convergent primers and found that circ_0002746, circ_0003052, circ_0004319 and circ_0012840, but not linear_0002746, linear_0003052, linear_0004319, and linear_0012840, were resistant to RNase R digestion. (**E**-**H**) Backsplice sites of circ_0002746, circ_0003052, circ_0004319 and circ_0012840 were confirmed by Sanger sequencing

To demonstrate the circular structure of the six candidate circRNAs, we used divergent primers and convergent primers to detect the resistance of circRNAs and linear RNAs to linear RNase R. The results showed that circ_0002746, circ_0004319, and circ_ 0012840 resisted linear RNase R digestion (Fig. 2A-D), but circ_0010571, circ_0007797 could not resist linear RNase R digestion (Fig. S1). Subsequently, the head-to-tail splice sites of circ_0002746, circ_0003052, circ_0004319, and circ_ 0012840 were also confirmed by through Sanger sequencing (Fig. 2E-H).

### Tissue expression and subcellular localization of candidate DE-circRNAs

**Fig. 3:**
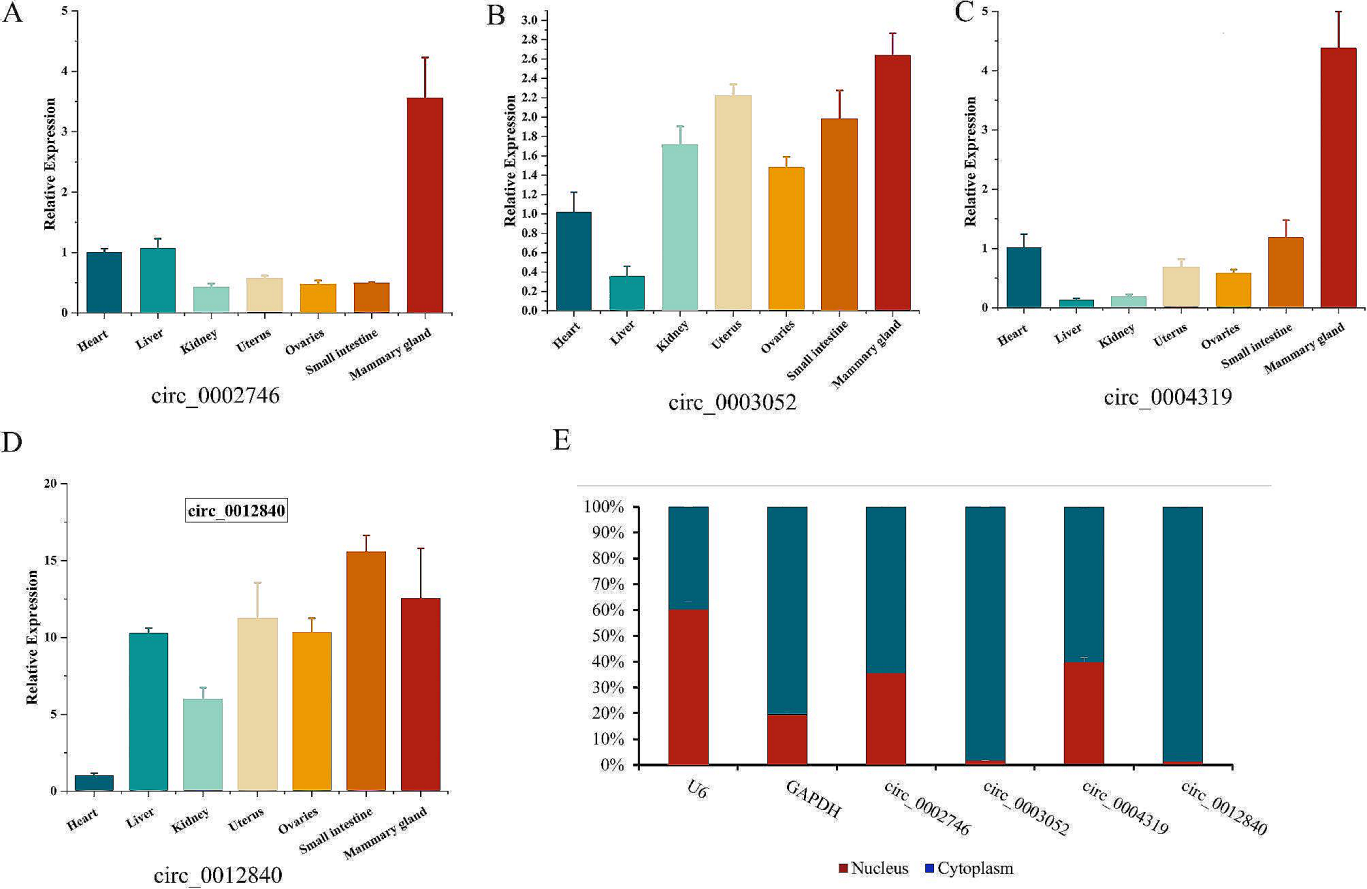
(**A**-**D**) The relative expression levels of circ_0002746, circ_0003052, circ_0004319 and circ_0012840 in different tissues of dairy cows, respectively. (**E**) The expression abundances of circ_0002746, circ_0003052, circ_0004319 and circ_0012840 in the cytoplasm and nucleus, with U6 and GAPDH as internal controls for nuclear and cytoplasm, respectively

To investigate the potential functions of circ_0002746, circ_0003052, circ_0004319 and circ_0012840 in the mammary glands of dairy cows, we used RT-qPCR to examine the expression levels of these circRNAs in various tissues of dairy cows. The results demonstrated that circ_0002746, circ_0003052, and circ_0004319 were highly expressed in mammary tissues compared with other tissues (Fig. 3A-C). In mammary tissue, the expression abundance of circ_ 0012840 was second only to that of the small intestine (Fig. 3D). To identify the specific locations where these circRNAs functioned, we isolated and examined the nucleus and cytoplasm of MECs using RT-qPCR. circ_0002746, circ_0003052, circ_0004319, and circ_0012840 were expressed at significantly higher levels in the cytoplasm than in the nucleus (Fig. 3E), suggesting that these four circRNAs may have potential regulatory functions on mammary gland development and lactation in dairy cows through the ceRNA network.

### Construction and screening of ceRNA networks for candidate circRNAs

**Fig. 4:**
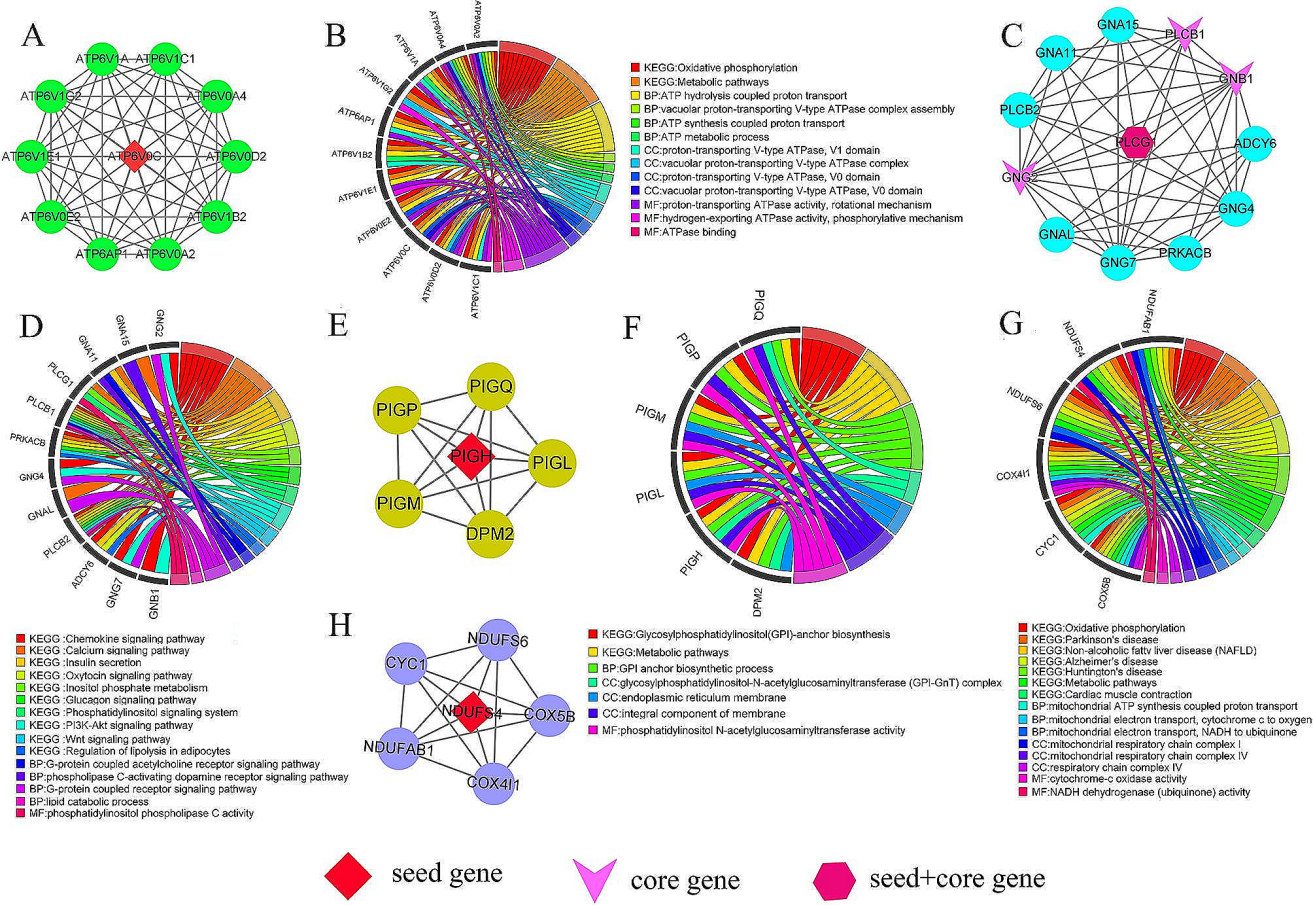
The crucial sub-networks and hub genes obtained by the cytoHubba and MCODE algorithms in Cytoscape. (**A**) (**C**) (**E**) (**H**) Four crucial sub-networks of the target gene screened from the *PPI* network. (**B**) (**D**) (**F**) (**G**) Enrichment analysis results of sub-networks (**A**), (**C**), (**E**), and (**H**) respectively

**Fig. 5:**
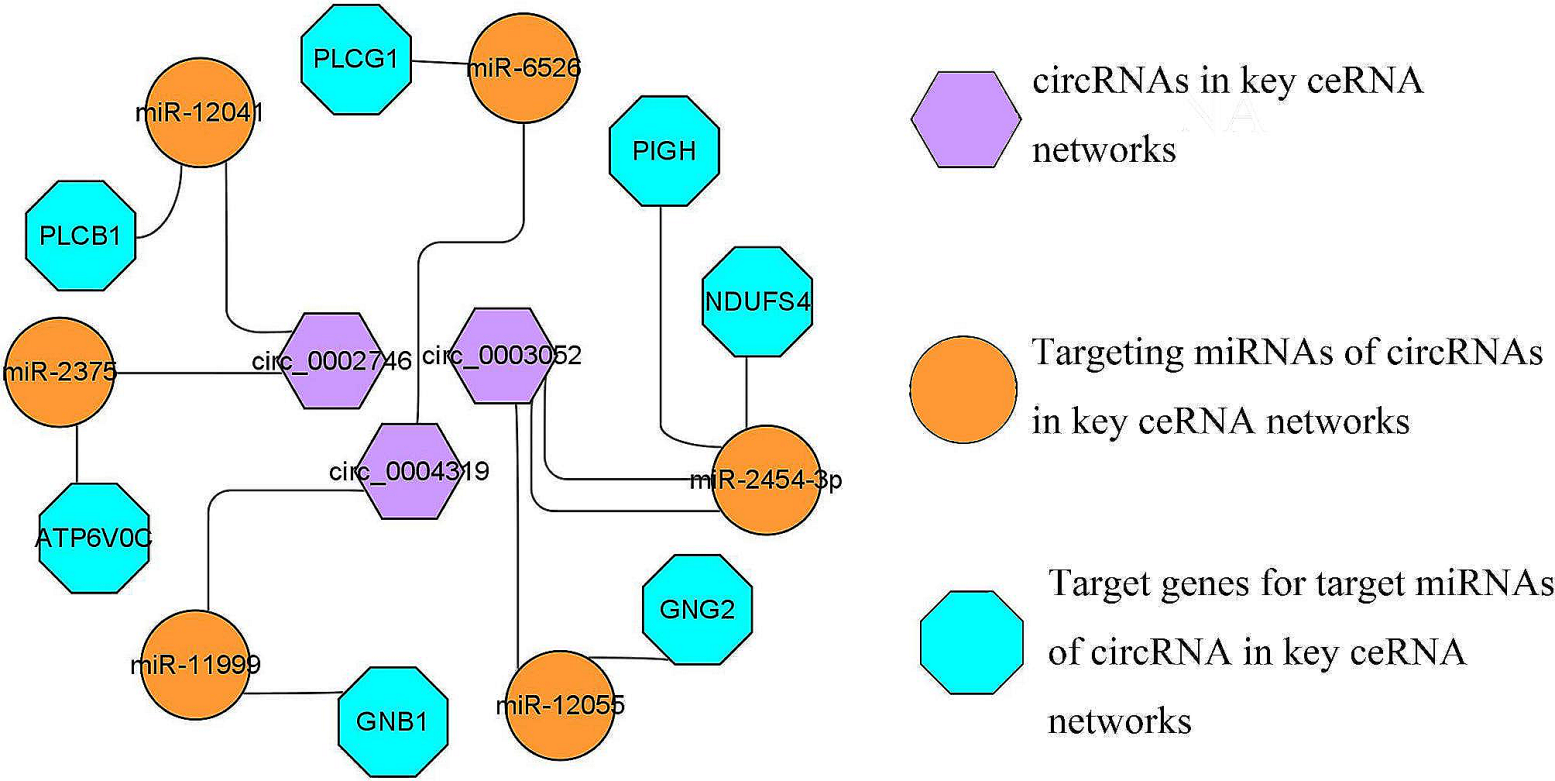
The candidate key ceRNA network regulating milk fat metabolism

We used the Targetscan (v7.2) [24] and miRanda (v3.3a) [25] software to predict circRNA/miRNA and miRNA/mRNA interactions and to screen the top 5 miRNAs that bind to each candidate circRNA (Table S4). We screened 372 target genes of the top 5 miRNAs bound by circRNAs according to the context+ ≤ − 0.20 and free energy ≤ − 20 of the miRNA/mRNA interaction relationship (Table S5). Although many target genes existed in the ceRNA network, we further screened the hub target genes. Hub genes are vital in biological processes, and their regulation often influences other genes within related pathways. We first identified four crucial sub-networks from the *PPI* network of target genes using the MCODE plugin in Cytoscape (Fig. 4A, C, E, H), and then, we performed gene ontologies (GO) and KEGG analysis to clarify the role of target genes in these subnetworks. Subnetwork 1’s main functions were focused on metabolic processes such as V-type ATPase for proton transport and ATP hydrolysis and synthesis, and the enriched pathways was mainly oxidative phosphorylation (Fig. 4B). Subnetwork 2 was enriched in G-protein coupled receptor signaling pathway, lipid catabolic process, phosphatidylinositol phospholipase C activity, and other GO terms. KEGG analysis was involved in lipid metabolism-related signaling pathways, such as calcium signaling pathway, insulin secretion, oxytocin signaling pathway, and phosphatidylinositol signaling system (Fig. 4D). Subnetwork 3’s functions were focused mainly on the glycosylphosphatidylinositol (GPI)-anchor biosynthetic process of biological process (BP). In cellular component (CC) and molecular function (MF), Subnetwork 3 was mainly involved in the synthesis of phosphatidylinositol-related proteins. The main enriched KEGG pathway was GPI-anchor biosynthesis (Fig. 4F). The target genes in subnetwork 4 performed their functions in the mitochondrial respiratory chain complex IV, and the main enriched pathway was also oxidative phosphorylation (Fig. 4G). We obtained seven hub genes from these four sub-networks: *GNB1, GNG2, PLCB1, PLCG1, ATP6V0C, NDUFS4*, and *PIGH* (Fig. 4A, C, E, H). These seven hub target genes compose key ceRNA network for exploring the mechanism of milk fat regulation in cows (Fig. 5).

## Discussion

WGCNA can combine gene expression with phenotypic data, making it more suitable for analyzing complex data. There are several applications for diverse omics data (e.g. transcriptomics, proteomics, and metabolomics) and various organisms (animal, plant, and microbial) [26–27]. WGCNA provides a solution to the multiple testing problems by reducing the size of large networks into a small number of hub nodes, allowing comparison of external traits with a limited number of variables. And can cluster genes with the same function or pathway to form functional modules [28]. Recognizing the core modules helps to annotate the results of systems biology scale experiments, thus adding valuable biological information. Therefore, we used WGCNA to construct a co-expression network of circRNAs from high- and low-MFP Holstein cows and found that the pink module was significantly and negatively correlated with MFP. Focal adhesion was the most significantly enriched pathway for circRNAs in this module, which connects downstream to the mitogen-activated protein kinase (MAPK) signaling pathway, thereby affecting lipid metabolism [29]. Among other pathways, the extracellular matrix (ECM) can regulate the proliferation, apoptosis, and polarity of MECs [30–32]. The AMPK signaling pathway acts as an energy sensor that regulates metabolism in the body and cells, including lipid metabolism [33]. In epithelial cells, tight junctions are essential for cell adhesion and prevent the lateral diffusion of lipids and proteins. Cholesterol and long-chain fatty acids are abundant in its plasma membrane. The adherens junction increases cholesterol levels in the plasma membrane to facilitate tight junction formation [34–35]. The enriched pathways suggest these circRNAs may have potential regulatory mechanisms for mammary gland development and milk fat metabolism. Finally, six candidate DE-circRNAs (circ_0010571, circ_0007797, circ_0002746, circ_0003052, circ_0004319, and circ_0012840) were screened from the pink module by combining differential expression analysis and WGCNA. The circular validation revealed that circ_0002746, circ_0003052, circ_0004319, and circ_0012840 belonged to circRNAs, which were the primary focus of this study.

Subcellular localization and tissue expression revealed that circ_0002746, circ_0003052, circ_0004319, and circ_0012840 were predominantly present in the cytoplasm and highly expressed in mammary tissue. These results provide strong evidence that the four DE-circRNAs to regulate mammary gland development and lactation by competitively binding miRNAs. In circRNA/miRNA and miRNA/mRNA interactions regulation, the target genes of circ_0002746, *PPARD*, *ELOVL2*, *LSS*, *ACAA2* and *PPARGC1A*, were associated with the regulation of lipid metabolism [36–37]. circ_0003052 as sponge of miR-2454-3p to regulate *SLC27A6* with high expression in adipose tissue, and inhibiting *SLC27A6* expression may significantly affect lipid metabolism pathways, including lipid biosynthesis, transport, and β-oxidation in mammary cells [38–39]. Among the miRNAs that interacted with circ_0004319, miR-11,999 exhibited the highest number of target genes, among which *ELOVL7*, *ACADS*, and *APC* were lipid metabolism-related candidate genes [40–41]. circ_0012840 and circ_0003052 interact with miR-7864 to regulate the expression of *PLA2G2E*, which promotes lipid accumulation in adipose tissue and liver [42]. circ_0012840 interacts with miR-214 and miR-761 to regulate *VPS4A*, an important regulator of endosomal cholesterol transport [43].

We conducted a *PPI* network analysis of target genes in the ceRNA regulatory network of four candidates DE-circRNAs and screened seven hub target genes from the *PPI* network. *GNB1* (guanine nucleotide-binding protein (G protein), beta polypeptide 1) encodes the Gβ subunit of a heterotrimeric G protein complex that includes Gα and Gγ subunits. This complex function can transduce multiple intracellular signaling cascades [44]. This gene promotes lipolysis in adipose tissue and, when expressed, increases blood glycerol levels [45]. *GNG2* (G protein subunit gamma 2) expression is positively correlated with adipocyte size [46], and its upregulation can directly activate PI3K I_B_, thereby activating the PI3K-Akt pathway [47]. This pathway is also regulated by the Gβγ subunits of the trimer G protein complex formed by *GNB1* and *GNG2* [48–49]. Gβ1γ2 produces phosphoinositol by stimulating phospholipase Cβ, activating MAPK and Akt [50]. This evidence suggests that *GNB1* and *GNG2* are involved in lipid metabolic pathways. *PLCB1* and *PLCG1* belong to the phospholipase C (PLC) gene. PLC protein—a key enzyme for metabolizing inositol lipids—plays a key role in multiple transmembrane signal transduction pathways that regulate various cellular processes, including cell proliferation and mobility [51]. *PLCB1* (phospholipase Cβ1) is involved in adipocyte differentiation [52]. *PLCG1* (phospholipase Cγ1) has two major lobes: one contains the active site that modifies lipids, and the other sits on top of the active site to prevent lipids from reaching it [53]. *PLCB1* and *PLCG1* are important candidate genes for fat deposition [54–55]. *ATP6V0C* and the genes in Subnetwork 2 are part of the vacuolar ATPase (V-ATPase), which is crucial in stimulating mitochondrial gluconeogenesis and insulin secretion in the body [56]. The tight binding between the lipid phosphatidylinositol 3,5-bisphosphate (PI(3,5)P2) and the membrane of V-ATPase can activate V-ATPase activity and proton pump [57–58]. Cholesterol depletion significantly affects V-ATPase activity and the initial transfer [59]. *ATP6V0C* affects glucose metabolism through phosphorylation during glycolysis [60]. *NDUFS4* is an auxiliary subunit of the mitochondrial membrane respiratory chain NADH dehydrogenase (complex I), and *NDUFS4* functions in the later stages of complex I assembly [61–62]. The NADH shuttle substantially maintains mitochondrial energy metabolism and glucose-induced insulin secretion [63]. The *PIGH* gene encodes an endoplasmic reticulum-associated protein involved in GPI anchor biosynthesis, which are glycolipids found in many blood cells that anchor proteins to the cell surface. The protein encoded by the PIGH gene is a subunit of GPI–N-acetylglucosamine transferase (GPI–GlcNAc transferase) that transfers GlcNAc to phosphatidylinositol (PI) lipids in endoplasmic reticulum cells [64]. In short, these hub target genes may be involved in energy metabolism, lipid metabolism, and mitochondrial function through ceRNA networks. The ceRNA network comprising the seven hub target genes as a key ceRNA network for exploring the mechanism of milk fat regulation in cows.

## Conclusion

We screened four DE-circRNAs using differential expression analysis, WGCNA, and circular validation. Tissue expression and subcellular localization suggested that these DE-circRNAs may have potential regulatory functions on mammary gland development and lactation in dairy cows through ceRNA networks. Thus, we constructed the ceRNA regulatory network of candidate DE-circRNAs and screened out the key ceRNA networks regulating milk fat metabolism, which helped us further explore the regulatory mechanism of milk fat metabolism. This study also provides new clues for molecular breeding of dairy cows.

## Methods

### Selection of experimental animals and sample preparation

Based on year-round dairy herd improvement (DHI) measurements at Nongkeng Helanshan Maosheng dairy farm, we screened 245 mid–late lactation Holstein cows with similar average daily milk yield (35.21–37.21 kg) and consistent feeding and management backgrounds (Table S6). We then screened 4 long-term high-MFP and 4 long-term low-MFP cows from 245 cows and aseptically collected fresh milk samples from each cow. One portion of each sample was sent to the testing center for DHI determination, whereas the other portion was placed in sterile water at 37 °C and returned to the laboratory for MEC isolation. The isolation, culture, and identification of MECs were completed during the early stage of our research group [65]. Cow milk MECs had the characteristic “pebble” morphology of epithelial cells (Fig. S2A), and S-shaped growth curve, which was consistent with cell growth (Fig. S2B), and the expression of epithelial cell-specific keratin 18 was positive (Figs. S2C, D). Besides, there was a significant difference in the triglyceride content of cow milk MECs of the high- and low-MFP groups (Fig. S2E), and the expression levels of the lipogenic genes *SCD*, *PPARγ*, and *FASN* were higher in the high-MFP group than in the low-MFP group (Fig. S3). The aforementioned sample preparation details are described in a recent paper [66].

### RNA-Seq library construction and sequencing

The sequencing in the present study belongs to the same batch as that in a recent study [66] and is, therefore, methodologically identical. Total RNA was extracted from cow milk MECs using the TRIzol method. RNA degradation and contamination were monitored on 1% agarose gels. RNA purity was checked using the NanoPhotometer® spectrophotometer (IMPLEN, CA, USA). RNA integrity was assessed using the RNA Nano 6000 Assay Kit of the Bioanalyzer 2100 System (Agilent Technologies, CA, USA). The 260/280 ratio of all samples ranged from 1.70 to 1.90, and the RNA Integrity Index (RIN) was ≥ 8.00. Sample RNA for circRNA sequencing was stripped of ribosomal RNA (Epicenter Ribozero™ rRNA Removal Kit, Epicentre, USA), and linear RNA was digested with RNase R (Epicentre, USA). Sequencing libraries were prepared according to the manufacturer’s instructions for the NEBNext® Ultra™ Directional RNA Library Prep Kit for Illumina® (NEB, USA). After passing the library inspection, Illumina PE150 sequencing was performed. After filtering the raw data, the obtained clean reads were aligned with the downloaded reference genome (https://bovinegenome.elsiklab.missouri.edu/downloads/ARS-UCD1.2) using the Bowtie2 software (v2.2.8).

### Identification and differential expression analysis of circRNAs

Identification of circRNAs and differential expression analysis were performed according to our previously described methods [66]. The circRNA was detected and identified using find_circ [67] and CIRI2 [68]. Transcripts per million (TPM) were used to normalize known and novel circRNAs in each sample [69]; normalized expression levels = (readCount × 1,000,000)/libsize (libsize is the sum of circRNA read counts). Differential expression analysis of transcript count matrices of high and low MFP in cow milk MECs was performed using the R package “DESeq2” [70]. The resulting *P*-value was adjusted using Benjamini and Hochberg’s approach for controlling the false discovery rate. Genes identified using DESeq2 with an adjusted *P-*value < 0.05 were designated as differentially expressed.

### Weighted gene co-expression network analysis (WGCNA)

WGCNA was used for network construction and identification of consensus modules. Weighted gene network construction requires the optimal selection of soft threshold power β to improve co-expression similarity and calculate the degree of adjacency. Therefore, the function “pickSoftThreshold” (based on the criterion of approximate scale-free topology) from the R package “WGCNA” [71] was used to pick out the optimal soft threshold power β. The function “blockwiseConsensusModules” was employ used to calculate the consensus topology overlap and produce consensus modules. Based on the WGCNA analysis parameters of Yang et al. [72], we set the following: power = soft threshold power β (when *r* = 0.80); modules containing 20 genes as a minimum number (minModuleSize = 20); the module detection sensitivity of 2 (*deepSplit* = 2); module merged cut height of 0.25 (mergeCutHeight = 0.25, i.e., merged into one module if the correlation coefficient of eigengenes within the module was greater than 0.75). To avoid rearrangement of eigengene within modules according to intramodular connectivity (KME), we set the following parameters: *minKMEtoStay* = 0, *maxBlockSize* = 10,000, and the remaining parameters followed the default values of the function. Subsequently, the co-expression network was built using the function “blockwiseModules”, with the following parameters: power = soft threshold power β; TOMType = “notations”; *minModuleSize* = 20; *mergeCutHeigh t* = 0.25; *maxBlockSize* = 20,000; *pamRespectsDendro* = FALSE; *verbose* = 3; the other parameters were set to default. This process produces co-expression modules that are significantly correlated with MFP. Finally, the GS and MM of the eigengenes in the consensus module were calculated using the function “corAndPvalue”, and the selection criteria for circRNAs were|MM| ≥ 0.90,|GS| ≥0.60.

### Circular structure verification

To verify the circular structure of circRNA, we designed divergent primers and convergent primers for each circRNA. First, circRNAs and linear RNAs were tested for resistance to RNase R. We performed RNase R treatment on the total RNA. A portion of the total RNAs was added with 5U/µg RNase R and 2 µl of 10× RNase R Reaction Buffer, whereas the other portion was added with an equal amount of RNase-free water and 2 µl of 10× RNase R Reaction Buffer. After incubating at 37 °C for 30 min. the RNase R-treated RNA was purified using the RNeasy MinElute Cleanup Kit (QIAGEN, Germany). The RNA was then reverse transcribed to cDNA, and the expression levels of circRNA and linear RNA were detected using PCR and reverse transcription-quantitative polymerase chain reaction (RT-qPCR). Subsequently, the head-to-tail splice sites of the circRNAs were identified through Sanger sequencing. The circular structure was verified using our previous verification method [66]. Table S7 lists the primer sequences used in the present study.

### Tissue expression and subcellular localization

The experimental methodology in this section is also consistent with our previous research methodology [66]. TRIzol reagent was used to extract the total RNA from tissues of the heart, liver, kidney, uterus, ovaries, small intestine, and mammary gland. RNA was isolated from the cell cytoplasm and nucleus using the Cytoplasmic and Nuclear RNA Purification kit (Norgen Biotek), and GAPDH and U6 were used as cytoplasmic and nuclear fractionation indicators, respectively. The first-strand cDNA was synthesized using the PrimeScript RT Reagent Kit with gDNA Eraser (Takara, Dalian, China). RT-qPCR was used to detect the expression of circRNAs in various tissues, the cytoplasm, and the nucleus.

### Target relationship prediction

CircRNA/miRNA and miRNA/mRNA interaction pairs were predicted using miRanda (v3.3a) [24] and TargetScan (v7.2) [25] software. circRNA/miRNA and miRNA/mRNA interactions were analyzed based on TargetScan’s Context + and miRanda’s Free Energy criterion.

### Analysis of hub genes in *PPI* networks

A *PPI* network analysis was performed following the methodology previously described by Yang et al. [72]. Protein network interactions were obtained using the Strings website (https://string-db.org/, v11.0), where a minimum interaction score of 0.90 deemed sufficient to obtain high-confidence protein network interactions. The MCODE plugin in Cytoscape was applied to identify critical subnetworks and seeds of nodes (or hub genes). The CytoHubba plugin in Cytoscape detects hub genes by four centrality methods—Degree, Edge Percolation Component (EPC), Maximum Cliff Centrality (MCC), and Maximum Neighborhood Component (MNC)—which are practical methods for identifying hub genes from *PPI* networks [73]. Subsequently, functional enrichment analysis was performed on the genes of key subnetworks. The function “enrichGO” was applied to the annotation of GO, including BP, MF, and CC. The function “enrichKEGG” was applied for the KEGG annotations to uncover relevant signaling pathways. All enrichment analysis results were visualized using the R package “ggplot2”.

### Electronic supplementary material

Below is the link to the electronic supplementary material.

Supplementary Material 1

Supplementary Material 2

Supplementary Material 3

Supplementary Material 4

Supplementary Material 5

Supplementary Material 6

Supplementary Material 7

Supplementary Material 8

Supplementary Material 9

Supplementary Material 10

## Data Availability

All data generated or analyzed in this study are included in this article (and its Supplementary Information file), and the datasets have been submitted to the SRA database with the accession number PRJNA730595. Data can be accessed at: https://www.ncbi.nlm.nih.gov/sra/PRJNA730595.
