# Supplementary material for: Mining key circRNA-associated-ceRNA networks for milk fat metabolism in cows with varying milk fat percentages
Source: BMC Genomics. 2024 Apr 1;25:323. doi: 10.1186/s12864-024-10252-y (PMC10983688; doi:10.1186/s12864-024-10252-y)
Supplement: Supplementary file 6 — Supplementary Material 6 [file 12864_2024_10252_MOESM6_ESM.docx]

**Table S6 Ingredient and nutrient composition in diet (dry matter basis) %**

| Ingredient | Content | Composition | Content |
| --- | --- | --- | --- |
| Alfalfa | 16.22 | Dry matter (kg) | 17.28 |
| Corn silage | 51.32 | Net energy for lactating cow (MJ/kg) | 7.76 |
| Tablet corn | 10.82 | Crude protein | 18.31 |
| Soybean meal | 10.82 | Neutral detergent fiber | 35.84 |
| Cotton meal | 5.41 | Acid detergent fiber | 21.85 |
| 10 % premix | 5.41 | Fat | 2.59 |
|  |  | Ca | 0.52 |
| Total | 100 | P | 0.35 |

Note: Each kg of premix contains 800 000 IU of V_A_, 200 000 IU of V_D_, 4 000 mg of V_E_, 1 200 mg of Cu, 6 000 mg of Fe, 4 000 mg of Mn, 4 000 mg of Zn, 40 mg of I, 40 mg of Co and 32 mg of Se.
