# Supplementary material for: Mining key circRNA-associated-ceRNA networks for milk fat metabolism in cows with varying milk fat percentages
Source: BMC Genomics. 2024 Apr 1;25:323. doi: 10.1186/s12864-024-10252-y (PMC10983688; doi:10.1186/s12864-024-10252-y)
Supplement: Supplementary file 7 — Supplementary Material 7 [file 12864_2024_10252_MOESM7_ESM.docx]

**Table S7. Divergent primers and convergent primers for circRNAs to verify their head-to-tail splicing**

| circRNA | Primer type | Primer sequences(5'→3') | Product length/bp | Annealing temperature/℃ |
| --- | --- | --- | --- | --- |
| circ_0002746 | Divergent | F:TCACTGGATCTACTGAGACTTT | 87 | 53.1 |
|  |  | R:CTTGATTTCTGAGCATCTTCCT |  | 55.7 |
| circ_0004319 | Divergent | F:GAAGAAAAGAGAAATAGCGG | 128 | 51.4 |
|  |  | R:TTGTGTTGCTCCAAAACACT |  | 53.7 |
| circ_0012840 | Divergent | F:TGGGAGATGCCGGATACATAG | 126 | 60.0 |
|  |  | R:CAGGTAGAGGGGCAACAAGGT |  | 60.7 |
| circ_0003052 | Divergent | F:AGCACGTTGCACATGTTTTA | 129 | 55.0 |
|  |  | R:GCTTGTAGCCACATCTTTCA |  | 53.6 |
| linear_0002746 | Convergent | F: TACTAAGCGGACCAGAACCT | 118 | 54.3 |
|  |  | R: GACGGGCTGATTAAAGTCTC |  | 54.0 |
| linear_0004319 | Convergent | F:TAGGTGTTGATGCTTTG | 91 | 43.3 |
|  |  | R:CTTTTCCGCTATTTCTC |  | 44.8 |
| linear_0003052 | Convergent | F: CCTTGGCCCATTGGTGTTACTA | 108 | 61.6 |
|  |  | R: TTTCCGCTGTGTCTCATCGTTT |  | 62.2 |
| linear_0012840 | Convergent | F: TTCATCTTGTCGTTCTCTCA | 120 | 50.6 |
|  |  | R: CTCCAAACACTATGTATCCG |  | 50.4 |
