# Supplementary material for: Mining key circRNA-associated-ceRNA networks for milk fat metabolism in cows with varying milk fat percentages
Source: BMC Genomics. 2024 Apr 1;25:323. doi: 10.1186/s12864-024-10252-y (PMC10983688; doi:10.1186/s12864-024-10252-y)
Supplement: Supplementary file 8 — Supplementary Material 8 [file 12864_2024_10252_MOESM8_ESM.docx]

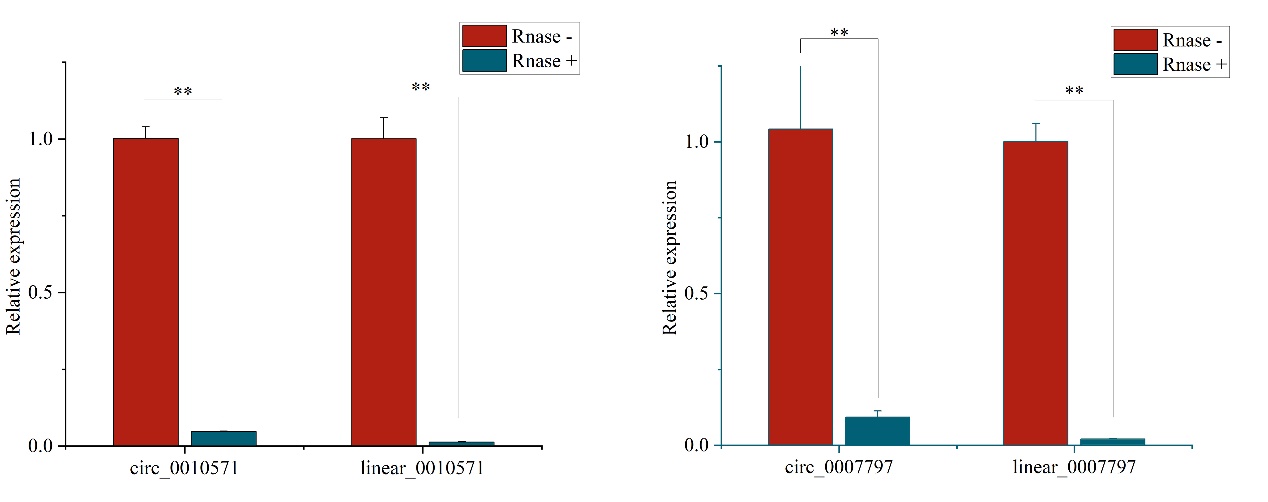


Figure S1: We identified by RT-qPCR using divergent and convergent primers and found that circ_0010571, linear_0010571 (left Figure), circ_0007797, and linear_0007797 (right Figure) were not resistant to RNase R digestion.
