# Supplementary figures and images for: Mining key circRNA-associated-ceRNA networks for milk fat metabolism in cows with varying milk fat percentages
Source: BMC Genomics. 2024 Apr 1;25:323. doi: 10.1186/s12864-024-10252-y (PMC10983688; doi:10.1186/s12864-024-10252-y)

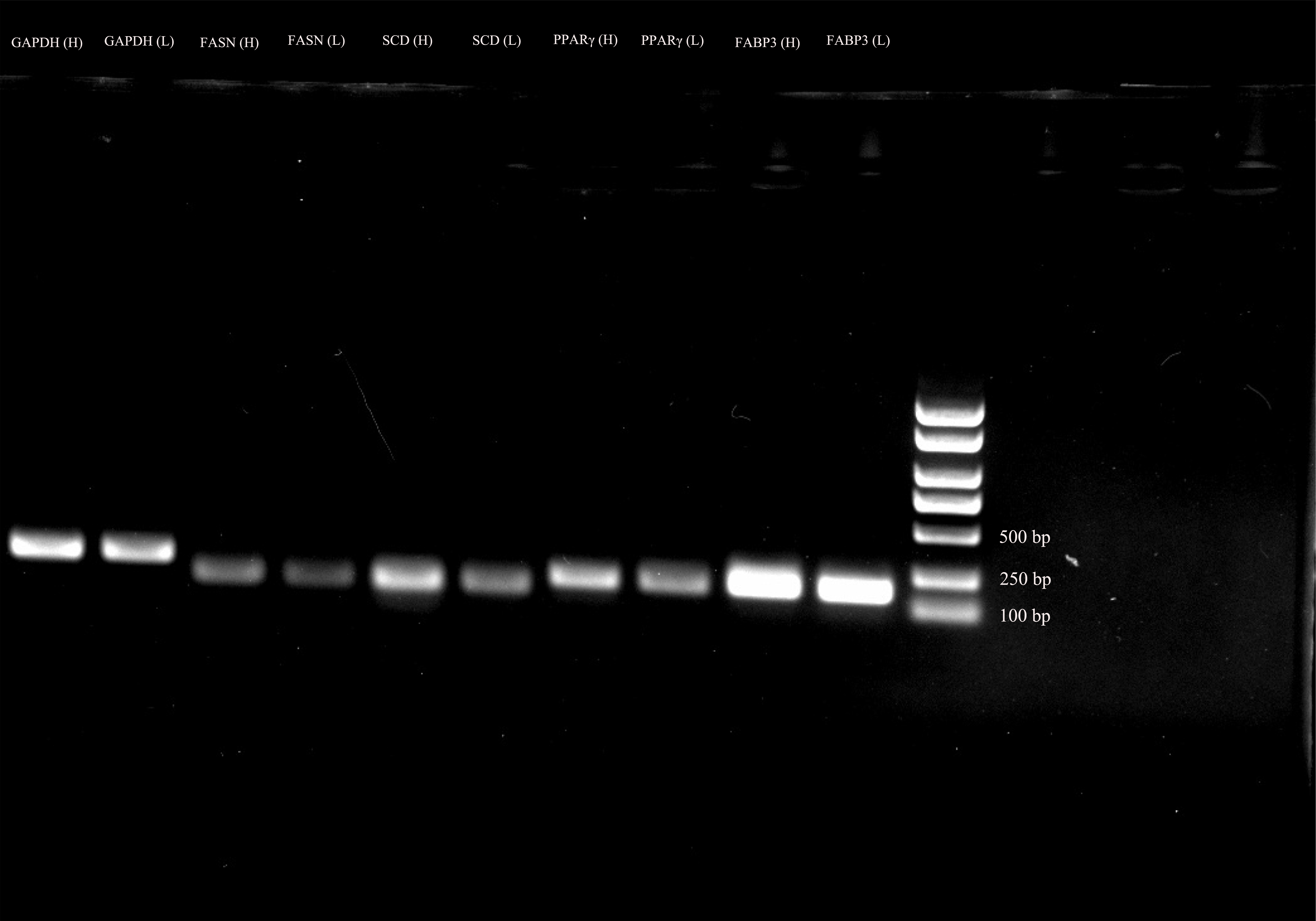

Supplement: Supplementary file 9 — Supplementary Material 9 [file 12864_2024_10252_MOESM9_ESM.jpg]

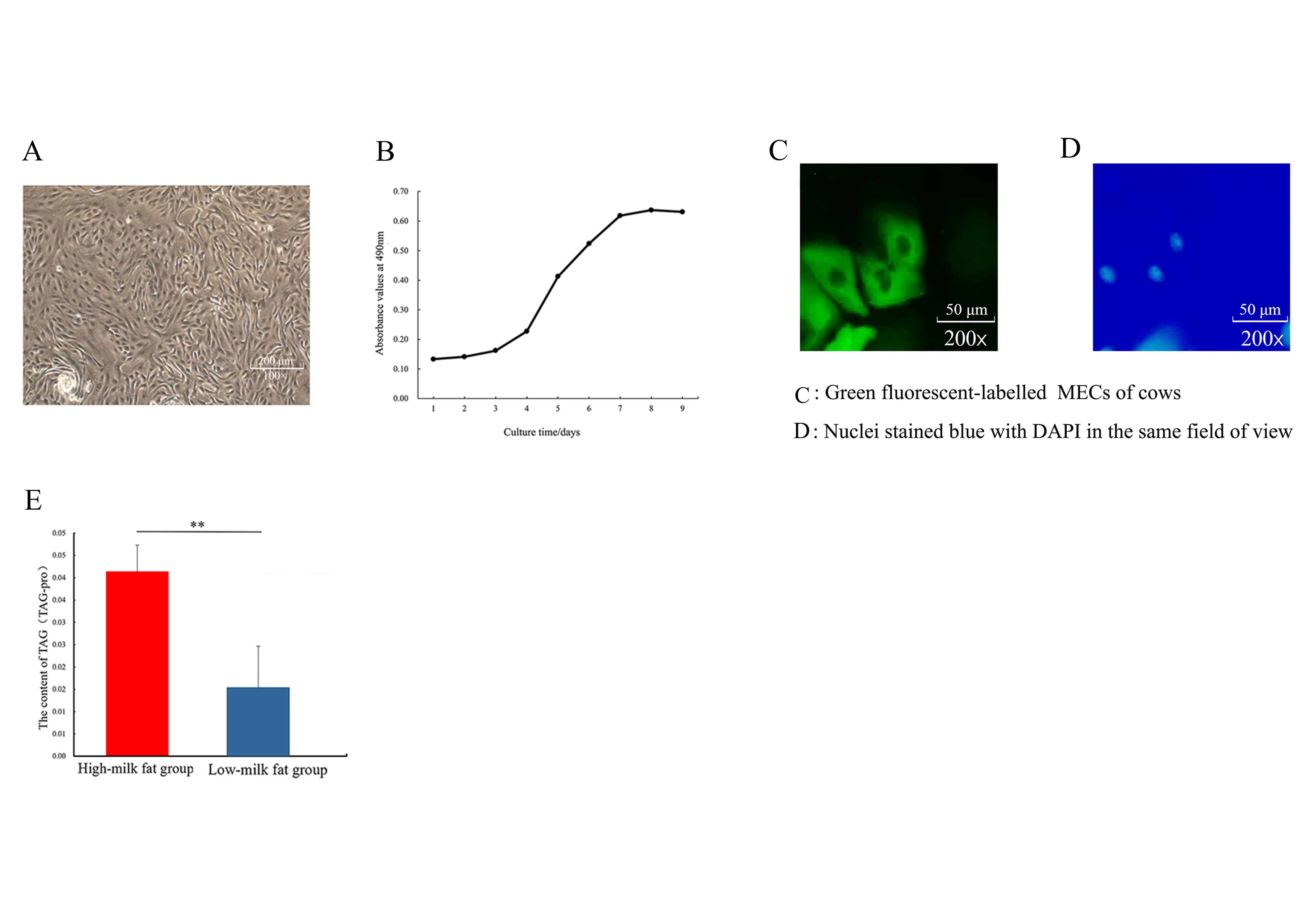

Supplement: Supplementary file 10 — Supplementary Material 10 [file 12864_2024_10252_MOESM10_ESM.jpg]
